# Problematic smartphone use and adolescent mental health: The mediating role of emotional growth mindset and the moderating role of left-behind experience

**DOI:** 10.1017/gmh.2026.10247

**Published:** 2026-06-15

**Authors:** Ruixin Mao, Xinyi Zhang, Yunxiang Xia, Lei Wang

**Affiliations:** Department of Psychology, Wuhan Sports University, Wuhan, China

**Keywords:** problematic smartphone use, mental health, senior high school students, emotional growth mindset, left-behind experience

## Abstract

This study aimed to identify latent profiles of mental health among Chinese senior high school students using latent profile analysis (LPA), and to examine the associations through which problematic smartphone use (PSU) is linked to mental health, focusing on the mediating role of emotional growth mindset (EGM) and the moderating role of left-behind experience (LBE). Using a dual-factor model of mental health as the conceptual basis for identifying mental health profiles, a cross-sectional survey was conducted of 34,831 students in Grades 10–12 in Tianmen, China. Validated scales were used to assess PSU, EGM, subjective well-being and depressive symptoms. LPA identified four distinct mental health profiles: very low depression–high well-being, low depression–moderate well-being, moderate depression–moderate well-being and high depression–low well-being. PSU was directly associated with poorer mental health and showed an indirect association through EGM. LBE moderated the first stage of the mediation pathway, that is, the PSU–EGM association, with a stronger effect observed among students with LBE.

## Impact statement

Millions of children and adolescents in low- and middle-income countries grow up with prolonged parental absence due to labour migration, displacement or other adversities, including left-behind children in rural China. For these young people, problematic smartphone use may pose mental health risks that cannot be addressed simply by reducing screen time. This study finds that problematic smartphone use is associated with poorer adolescent mental health, and this association is statistically consistent with a reduced belief that emotions can be changed and managed. This finding has practical value because such beliefs are teachable. In resource-limited settings where parental supervision is often inconsistent, schools may be able to support students by incorporating emotion mindset training into existing mental health education. This approach may be especially useful for left-behind and other separated adolescents, who typically have fewer opportunities for daily emotional guidance from parents. By identifying a potentially modifiable psychological factor, the study offers a preliminary basis for future research to explore low-cost, school-based programmes that may help adolescents in LMICs develop healthier relationships with digital technology and strengthen their emotional resilience.

## Introduction

By December 2025, China had 1.125 billion mobile internet users, with an internet penetration rate of 80.1% (CNNIC, 2026). As such, the implications of digital technology use for adolescent mental health have become an important public mental health concern. Although many cross-sectional studies report negative associations between digital technology use and adolescent mental health (Coyne et al., 2019; Monks et al., 2019; Busch and McCarthy, 2021), recent evidence is mixed. For example, the National Academies of Sciences, Engineering and Medicine (NASEM) reported that current evidence does not yet provide robust support for a consistent association between social media use and adolescent health outcomes (NASEM, 2024; Mansfield et al., 2025). These inconsistent findings suggest that the mental health correlates of digital technology use might depend on use patterns, individual susceptibility and contextual conditions, instead of exposure alone (Odgers and Jensen, 2020; Sanchez and Jenkins, 2024). Accordingly, research needs to move beyond simple measures of screen time and examine specific forms of problematic use, relevant psychological correlates and vulnerable subgroups (NASEM, 2024).

Problematic smartphone use (PSU) might be associated with adolescents’ lower sense of autonomy, competence and relatedness. Specifically, the compulsive and dysregulated features of PSU (e.g., loss of control over use and withdrawal-like experiences when access is restricted) might make adolescents feel less able to regulate their own behaviour and less engaged in self-directed action. Frequent smartphone checking, distraction and disrupted attention might also be linked to reduced academic engagement, poorer task performance and a lower sense of competence (Coyne et al., 2019; Paterna et al., 2024). PSU might also coincide with fewer opportunities for high-quality face-to-face interaction, poorer offline relationship quality and stronger feelings of social disconnection (Coyne et al., 2019; Paterna et al., 2024). Consistent with this evidence, PSU has been associated with higher levels of anxiety and depressive symptoms, and lower subjective well-being (Coyne et al., 2019; Mahapatra, 2019; Wang et al., 2025). However, most previous studies have treated mental health as a single dimension, which might obfuscate important heterogeneity in adolescents’ psychological functioning.

To address this limitation, the present cross-sectional study adopts a dual-factor model of mental health (Wang and Zhang, 2011), which integrates psychopathological symptoms with positive psychological functioning. Within this framework, depressive symptoms and subjective well-being are related but distinct dimensions, and their combinations might form qualitatively different mental health profiles. Previous studies have used these two indicators to identify groups such as complete mental health, vulnerable, symptomatic but content and troubled groups (Suldo and Shaffer, 2008). Recent research on Chinese adolescent and student populations also supports the usefulness of this approach for identifying meaningful heterogeneity in mental health (Chen et al., 2022; Sun et al., 2025). Building on this evidence, this study selected depressive symptoms and subjective well-being as indicators for latent profile analysis (LPA) to identify mental health profiles among senior high school students. Unlike variable-centred methods, which assume uniform associations, LPA is a person-centred approach identifying subgroups on the basis of individuals’ depression and well-being scores. This approach allows mental health profiles to be derived empirically without imposing arbitrary cut-offs. Examining PSU across these profiles could therefore provide a more nuanced understanding of adolescent adjustment.

Beyond identifying heterogeneous mental health profiles, it is also important to clarify the psychological factors involved in the PSU–mental health association. Cognitive resources for higher-order processing are limited, and excessive cognitive demands might impair functions such as emotion regulation (Sweller, 2010). Related research also suggests that cognitive control and emotion regulation draw on a shared and finite pool of resources, such that resource depletion might weaken regulatory capacity (Baumeister et al., 1998). PSU might create a cognitively demanding environment through frequent attentional shifts and multitasking (Wickord and Quaiser-Pohl, 2025), thereby being associated with greater reliance on maladaptive strategies such as avoidance, rather than on more adaptive strategies such as cognitive reappraisal (Elhai et al., 2018). Over time, this process might be associated with less belief in emotional controllability (Ford and Gross, 2018) and less of an emotional growth mindset (EGM), defined as the belief that emotions can be changed through effort. An EGM is associated with greater well-being and lower depression levels (Kneeland and Dovidio, 2020; Skymba et al., 2022), and might be relevant to adolescent mental health through enhanced psychological capital (Xiong et al., 2020; Zhang et al., 2023).

Family context might further influence adolescents’ susceptibility to the mental health correlates of PSU. The family context is an important setting with respect to adolescent psychosocial development, and recent evidence from rural China indicates that greater involvement of the father is associated with lower depression and anxiety levels among children and adolescents (Jiang et al., 2024). Thus, left-behind adolescents represent an important group for consideration. In China, left-behind adolescents are typically those whose parents migrate for work, leaving them to live with grandparents or other relatives for extended periods (Hu and Xiao, 2023). Although this phenomenon is particularly prominent in China, prolonged parental absence is not unique to this context. In many low- and middle-income countries, and in other resource-limited settings, children and adolescents might experience separation from one or both parents owing to labour migration, divorce, parental incarceration, forced displacement or other social adversities, all of which could weaken family support. Evidence from displaced populations in low- and middle-income countries also suggests that children and adolescents might face distinct challenges related to prolonged separation from caregivers, disrupted social support systems and psychological distress (Dickson et al., 2024). Therefore, prolonged parental absence might be associated with emotional neglect and less support for these adolescents (Fellmeth et al., 2018). Unmet emotional needs might increase adolescents’ motivation to seek compensation through online engagement, thereby being associated with greater susceptibility to PSU (Kardefelt-Winther, 2014). Moreover, limited offline support might be associated with fewer resources for coping with attentional and emotional demands related to PSU, which might make difficulties with emotion regulation more likely (Zhen et al., 2020).

Taken together, existing evidence suggests that PSU is associated with poorer adolescent mental health, but less is known about how this association manifests across distinct combinations of depressive symptoms and subjective well-being. It also remains unclear whether an EGM is involved in PSU–depressive symptom associations and subjective well-being, and whether the PSU–EGM association differs according to left-behind experience (LBE). Based on previous evidence, the present cross-sectional study proposed the following hypotheses: H1, senior high school students would be classified into four latent mental health profiles; H2, PSU would differ significantly across these profiles; H3, EGM would statistically mediate the associations of PSU with depressive symptoms and subjective well-being; and H4, left-behind status would moderate the PSU–EGM association, with a stronger negative association expected among students with LBE than those without such experience.

## Methods

### Participants

Data were collected between early April and early June 2025 using cluster sampling, with class as the primary sampling unit. In total, 35,911 questionnaires were distributed to students in Grades 10–12 from several senior high schools in Tianmen, China. After removing incomplete and invalid responses, including those with missing key variables or patterned responding, 34,831 valid responses remained (valid response rate = 97.0%). Among the 34,831 participants, 22,063 (63.3%) reported LBE. The sample included 20,095 male students (57.7%) and 14,736 female students (42.3%). Most participants attended public schools (*n* = 22,993, 66.0%). The grade distribution was relatively balanced: 13,870 students were in Grade 10 (39.8%), 11,773 in Grade 11 (33.8%) and 9,188 in Grade 12 (26.4%). Regarding family structure, most participants came from intact families (*n* = 30,403, 87.3%), followed by blended families (*n* = 2,324, 6.7%), single-parent families (*n* = 1,943, 5.6%) and orphanages (*n* = 161, 0.5%) (Supplementary Table S1).

### Measures

#### Problematic Smartphone Use Scale–Short (PMPUS-S)

Problematic smartphone use was assessed using the eight-item PMPUS-S, which measures compulsive and dysregulated patterns of smartphone use associated with adverse consequences (example item: ‘I feel anxious when unable to receive messages/calls due to an absent phone’; Zhou et al., 2023). Items were rated on a five-point Likert scale ranging from 1 (*strongly disagree*) to 5 (*strongly agree*), with higher scores indicating more severe PSU. The scale showed excellent internal consistency in our sample (Cronbach’s *α* = 0.93). The Chinese version of the PMPUS-S has been validated among adolescents, demonstrating good reliability and validity and measurement invariance across age and gender (Zhou et al., 2023).

#### World Health Organization Well-being Index (WHO-5)

Subjective psychological well-being was assessed using the five-item WHO-5, which captures positive mood, vitality and general psychological well-being over the preceding 2 weeks (example item: ‘I have felt cheerful and in good spirits’; World Health Organization, 1998). Items were rated on a six-point scale ranging from 0 (*all of the time*) to 5 (*never*), and were reverse-coded, with higher scores indicating greater well-being. The scale showed excellent internal consistency in our sample (Cronbach’s *α* = 0.91). The Chinese version of the WHO-5 has been validated among young Chinese adults, demonstrating good structural validity, internal consistency and measurement invariance (Yang et al., 2023).

#### Kutcher Adolescent Depression Scale (KADS-11)

Depressive symptoms were assessed using the KADS-11, which comprises 11 items covering emotional, cognitive and somatic aspects of depression (example item: ‘feeling irritable’; Zhou et al., 2015). Items were rated on a four-point scale ranging from 0 (*rarely*) to 3 (*all of the time*), with higher scores indicating more severe depressive symptoms. The scale showed excellent internal consistency in our sample (Cronbach’s *α* = 0.90). The Chinese version of the KADS-11 has been validated in a large sample of Chinese adolescents, demonstrating good convergent validity, internal consistency, test–retest reliability and diagnostic accuracy (Zhou et al., 2015).

#### Theories of Emotion Scale (TOE)

EGM was assessed using the TOE. EGM refers to the belief that emotions can be changed and regulated through effort. The scale, developed by Schroder et al. (2015), comprises four items (example item: ‘Everyone can learn to control their emotions’; Schroder et al., 2015). Items were rated on a six-point scale ranging from 1 (*strongly disagree*) to 6 (*strongly agree*), with items 3 and 4 reverse-coded. Higher scores indicated a stronger EGM. The scale showed acceptable internal consistency in our sample (Cronbach’s *α* = 0.77). The TOE has been validated in Western adolescent and young adult samples, demonstrating sound psychometric properties (Schroder et al., 2015). However, a dedicated validation study among Chinese adolescents is currently lacking; this is acknowledged as a limitation.

#### LBE

LBE was assessed using a single dichotomous item. The item identified whether participants had experienced the prolonged absence (> 6 months) of important family members, particularly their father and/or mother, before the age of 6 years. The item asked: ‘Before the age of six, were important family members, such as your father and/or mother, frequently absent from your life for more than six months?’ Participants who answered ‘yes’ were coded as having LBE (LBE = 1), whereas those who answered ‘no’ were coded as not having LBE (LBE = 0). This single-item measure has been used in large-scale studies of left-behind children in China (Fellmeth et al., 2018).

### Data analysis

LPA was conducted in Mplus 8.0 (Muthén and Muthén, 2017) to identify latent classes based on well-being and depressive symptoms. Specifically, the five items of the WHO-5 Well-Being Index and the 11 items of the KADS-11 were entered as item-level indicators, resulting in 16 indicators in total. The optimal number of classes was determined using several statistical and substantive criteria, including information criteria, entropy, likelihood ratio tests and substantive interpretability (Nylund et al., 2007). Lower Akaike information criterion (AIC), Bayesian information criterion (BIC) and sample-size-adjusted Bayesian information criterion (aBIC) values were taken to indicate better model fit. Higher entropy values were considered indicative of better classification precision, with values ≥0.80 considered acceptable. Significant values for the Lo–Mendell–Rubin-adjusted likelihood ratio test (LMRT) and the bootstrap likelihood ratio test (BLRT) indicated that a *k*-class model provided a better fit than a *k* − 1-class model. Class size was also considered, with a preferred minimum proportion of 5% in each class.

After the optimal class solution had been identified, the Bolck–Croon–Hagenaars (BCH) procedure in Mplus was used to compare PSU and EGM across the latent classes. The BCH method accounts for classification uncertainty by weighting each participant’s contribution according to their posterior class probabilities. This approach is more appropriate than conventional analysis of variance, which treats class membership as fixed and, therefore, ignores uncertainty in class assignment (Asparouhov and Muthén, 2014).

Descriptive statistics and correlation analyses were conducted using SPSS 26.0 (IBM Corp., Armonk, NY, USA). Mediation and moderated mediation analyses were performed using the PROCESS macro, specifically Models 4 and 7. Indirect effects and their confidence intervals (CIs) were estimated using 5,000 bootstrap samples. All models controlled for age, gender, grade, school type, family structure, and LBE. LBE was included as a covariate in Model 4 and as the moderator in Model 7.

## Results

### Common method bias test

Harman’s single-factor test was used to assess potential common method bias. Seven factors with eigenvalues >1.0 were extracted. The first factor explained 31% of the total variance, which was below the conventional threshold of 40%. According to Harman’s single-factor test, substantial common method bias was unlikely. However, because all variables were measured using self-report questionnaires from the same source and at the same time point, common method variance cannot be completely ruled out. This issue should therefore be considered as a potential limitation.

### LPA of senior high school students’ mental health

As the number of latent classes increased, the AIC, BIC and aBIC values decreased, indicating improved model fit. The four-class model showed good classification precision (entropy value = 0.955). The BLRT and LMRT were significant (both *p* < 0.001). The average posterior probabilities for the four latent classes were 0.91–0.97, indicating good class separation. The class proportions were 34.44%, 52.59%, 9.37% and 3.60%. Although the smallest class accounted for less than the conventional 5% threshold, it was retained because it showed a distinct psychological profile, and the solution remained stable across multiple random starts. Overall, the model fit indices, classification quality, class separation and theoretical interpretability supported the four-class model as the optimal solution (Table 1).

**Table 1.** Summary of fit indices for latent profile analysis Table 1. long description.

No. of classes	AIC	BIC	aBIC	LMRT(*p*)	BLRT(*p*)	Entropy	Class probabilities (%)
1	1,448,420.495	1,448,691.159	1,448,589.463				1
2	1,278,533.595	1,278,948.05	1,278,792.328	<0.001	<0.001	0.927	33.99/66.01
3	1,218,452.957	1,219,011.202	1,218,801.454	<0.001	<0.001	0.912	48.16/38.84/13.00
**4**	**1,172,222.08**	**1,172,924.115**	**1,172,660.342**	**<0.001**	**<0.001**	**0.955**	**34.44/52.59/9.37/3.60**

*Note:* The bold row indicates the best-fitting model based on statistical indices.

**Figure 1.**
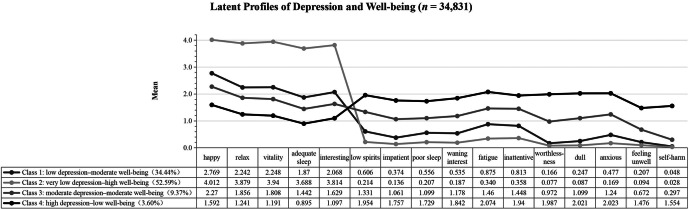
Latent profiles of depression and well-being (*n* = 34,831). The *x*-axis comprises the WHO-5 and KADS-11 items, with the WHO-5 items further described in Supplementary Scale 1. Over the past two weeks, the frequency with which participants felt happy, relaxed, vitality, adequate sleep and that each day was filled with interesting things, where 0 = *all the time*, 1 = *most of the time*, 2 = *more than half the time*, 3 = *less than half the time*, 4 = *sometimes* and 5 = *never.* The KADS-11 items are further described in Supplementary Scale 2. Frequency over the past month of participants feeling low spirits, impatient, poor sleep, waning interest, fatigue, inattentive, worthlessness, dull, anxious, feeling unwell and thoughts or actions of harming oneself, where 0 = *Rarely*, 1 = *Often*, 2 = *Most of the time*, 3 = *All the time.* Figure 1. long description.

The LPA identified four distinct mental health profiles based on depressive symptoms and well-being (Figure 1):

Class 1 accounted for 34.44% of the sample and was characterised by low depressive symptoms (mean [M] = 0.45, standard deviation [SD] = 0.23; range: 0–3) and moderate well-being (*M* = 2.22, SD = 0.67; range: 0–5). This profile was labelled low depression–moderate well-being.

Class 2 accounted for 52.59% of the sample and was characterised by very low depressive symptoms (*M* = 0.17, SD = 0.21; range: 0–3) and high well-being (*M* = 3.87, SD = 0.58; range: 0–5). This profile represented the most favourable mental health pattern and was labelled very low depression–high well-being.

Class 3 accounted for 9.37% of the sample and was characterised by moderate depressive symptoms (*M* = 1.08, SD = 0.24; range: 0–3) and moderate well-being (*M* = 1.80, SD = 0.82; range: 0–5). This profile reflected elevated depressive symptoms alongside a moderate level of well-being, suggesting a mixed pattern of psychological distress and preserved positive functioning. It was labelled moderate depression–moderate well-being.

Class 4 accounted for 3.60% of the sample and was characterised by high depressive symptoms (*M* = 1.86, SD = 0.39; range: 0–3) and low well-being (*M* = 1.20, SD = 0.86; range: 0–5). This profile represented the least favourable mental health pattern and was labelled high depression–low well-being.

Because depressive symptoms and well-being were used as indicators of latent classes, inferential tests were not conducted for these variables. To examine whether PSU and EGM differed across the four latent classes, the BCH procedure was used in Mplus. Unlike conventional analysis of variance, which treats class membership as fixed, the BCH method accounts for classification uncertainty by weighting each participant’s contribution according to their posterior class probabilities. This approach is therefore more appropriate for comparing auxiliary variables across latent classes, reducing the risk of biased standard errors and inflated Type I error rates (Asparouhov and Muthén, 2014).

The overall BCH tests were significant for both PSU (*χ* ^2^[3] = 10,729.47, *p* < 0.001) and EGM (*χ* ^2^[3] = 4,326.45, *p* < 0.001). Post hoc pairwise comparisons showed that PSU increased across the profiles in the following order: Class 2, very low depression–high well-being (*M* = 1.82, SE = 0.007); Class 1, low depression–moderate well-being (*M* = 2.36, SE = 0.010); Class 3, moderate depression–moderate well-being (*M* = 3.02, SE = 0.012); and Class 4, high depression–low well-being (*M* = 3.57, SE = 0.025). By contrast, EGM decreased across the profiles in the following order: Class 2, very low depression–high well-being (*M* = 4.10, SE = 0.008); Class 1, low depression–moderate well-being (*M* = 3.83, SE = 0.009); Class 3, moderate depression–moderate well-being (*M* = 3.41, SE = 0.010); and Class 4, high depression–low well-being (*M* = 2.95, SE = 0.023). All pairwise comparisons were statistically significant (all *p* < 0.001) (Table 2).

**Table 2.** Differences in problematic smartphone use and emotional growth mindset across latent classes (*M* ± SE) Table 2. long description.

Measure	Class 1 (low depression–moderate well-being)	Class 2 (very low depression–high well-being)	Class 3 (moderate depression–moderate well-being)	Class 4 (high depression–low well-being)	Overall *χ* ^2^ (*df* = 3)	Post hoc comparisons (all *P* < 0.001)
Problematic smartphone use	2.36 ± 0.01	1.82 ± 0.007	3.02 ± 0.012	3.57 ± 0.025	10,729.47***	C2 < C1 < C3 < C4
Emotional growth mindset	3.83 ± 0.009	4.10 ± 0.008	3.41 ± 0.01	2.95 ± 0.023	4,326.445***	C4 < C3 < C1 < C2

*Note:* ***p < 0.001. The same applies below.

### Correlation analysis

Bivariate correlation analyses were conducted among PSU, EGM, depressive symptoms and well-being. All pairwise correlations were statistically significant (Supplementary Table S2).

### Moderated mediation model

**Figure 2.**
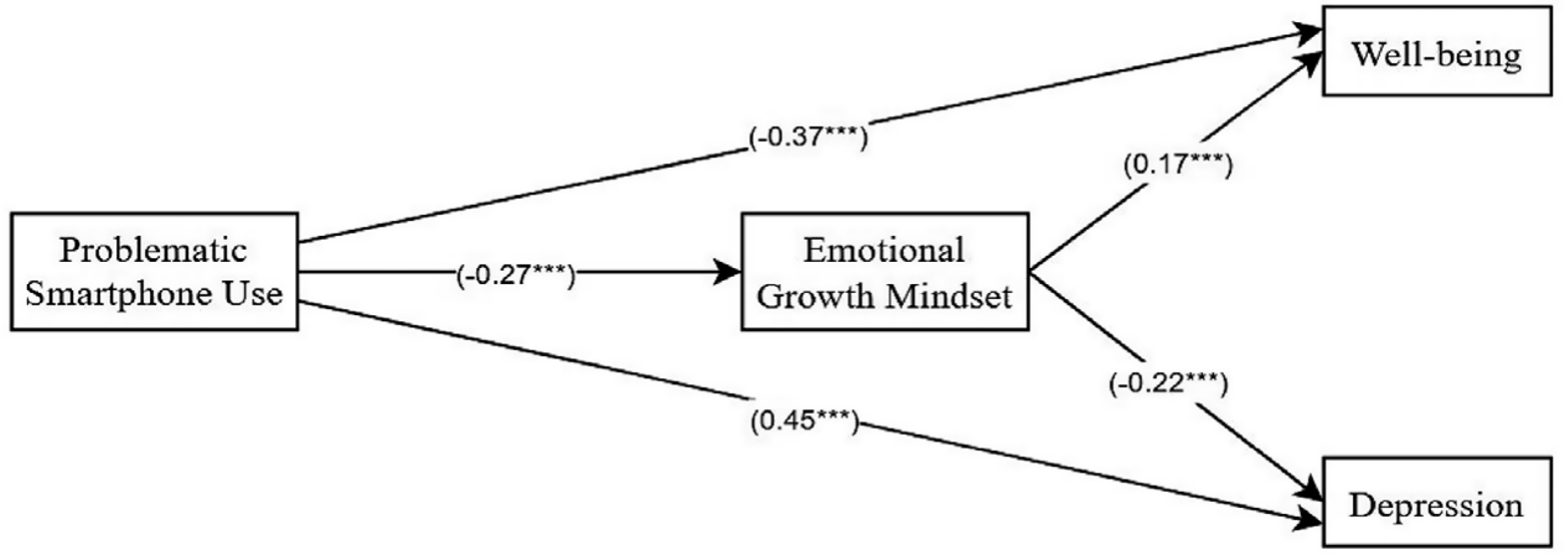
The mediating role of emotional growth mindset in the relationship between problematic smartphone use and well-being/depression. All coefficients in the figure are standardized values. The partial mediation effect of emotional growth mindset accounts for 10.85% of the total effect of problematic smartphone use on well-being and 11.68% of the total effect on depression. Figure 2 long description.

First, the mediating role of EGM was examined using Model 4 of the PROCESS macro in SPSS (Hayes, 2013). The results are presented in Figure 2. After controlling for age, gender, grade, school type, family structure and LBE, the indirect effects of PSU on well-being and depressive symptoms through EGM were statistically significant. The indirect effects were − 0.032 for well-being and 0.041 for depressive symptoms, accounting for 10.85% and 11.68% of the corresponding total effects, respectively. On including EGM in the model, PSU remained significantly associated with lower well-being (*β* = −0.37, *B* = −0.26) and more severe depressive symptoms (*β* = 0.45, *B* = 0.31). These findings suggest that EGM partially mediated the associations of PSU with well-being and depressive symptoms among senior high school students.

Model 7 of the PROCESS macro was then used to examine whether LBE moderated the first stage of the mediation pathway, that is, the PSU–EGM association. The results are presented in Table 3. PSU was negatively associated with EGM (*β* = −0.12, *p* < 0.001). The PSU–LBE interaction was also significant and negative (*β* = −0.02, *p* < 0.001), indicating that LBE significantly moderated the PSU–EGM association.

**Table 3.** Results of the moderated mediation analysis Table 3. long description.

Predictor	Emotional growth mindset			Well-being			Depression		
	*β*	SE	*t*	*β*	SE	*t*	*β*	SE	*t*
Problematic smartphone use	−0.12	0.002	−51.33^***^	−0.26	0.003	−74.72^***^	0.31	0.003	98.60^***^
Emotional growth mindset				0.26	0.008	33.61^***^	−0.34	0.007	−48.72^***^
Left-behind experience	0.35	0.04	8.67^***^						
PSU × Left-behind experience	−0.02	0.005	−3.41^***^						
*F*			461.97			1,652.26			2,826.54
*R* ^2^			0.09			0.22			0.33

***p < 0.001. The same applies below.

**Figure 3.**
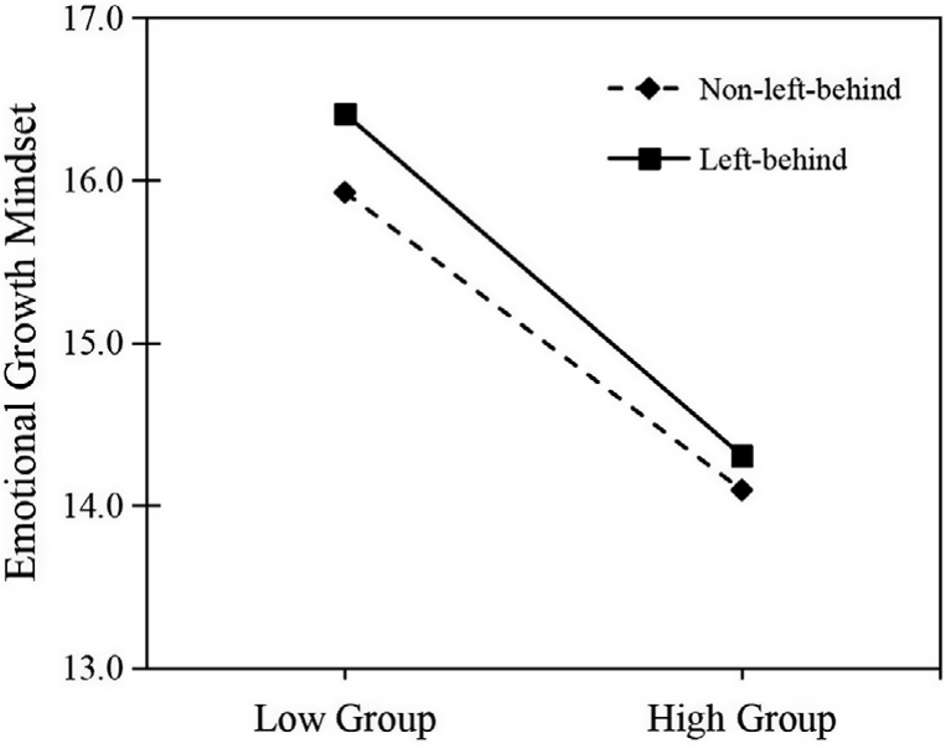
The moderating effect of left-behind experience on the relationship. Between problematic smartphone use and emotional growth mindset. Figure 3 long description.

To further probe the PSU–LBE interaction, simple slope analyses were conducted (Figure 3). Among students with LBE, PSU was negatively associated with EGM (*β*_simple = −0.13, p < 0.001). Among students without LBE, this association was also significant but slightly weaker (*β*_simple = −0.11, *p* < 0.001). These findings indicate that the negative PSU–EGM association was stronger among students with LBE than among those without such experience.

Further moderated mediation model analyses examined conditional indirect effects by left-behind status. Among students without LBE, the indirect effects of PSU on well-being and depressive symptoms through EGM were − 0.03 (95% CI: [−0.032, −0.026]) and 0.04 (95% CI: [0.034, 0.041]), accounting for 10.90% and 12.00% of the corresponding total effects, respectively. Among students with LBE, the corresponding indirect effects were − 0.03 (95% CI: [−0.036, −0.031]) and 0.04 (95% CI: [0.040, 0.046]), accounting for 12.53% and 13.79% of the corresponding total effects, respectively. These results suggest that the indirect associations of PSU with well-being and depressive symptoms through EGM were slightly stronger among students with LBE than among those without such experience.

## Discussion

This study identified four distinct latent mental health profiles among senior high school students, supporting H1 and providing further evidence for the utility of the dual-factor model of mental health in adolescent populations (Wang and Zhang, 2011; Chen et al., 2022). Specifically, the profiles were labelled very low depression–high well-being (Class 2), low depression–moderate well-being (Class 1), moderate depression–moderate well-being (Class 3) and high depression–low well-being (Class 4). These findings suggest that adolescent mental health cannot be adequately captured by a single symptom-based continuum. Rather, depressive symptoms and subjective well-being might coexist in different configurations, which has important implications for assessment and future intervention-oriented research.

Notably, the two intermediate profiles further illustrate why a dual-factor perspective is useful for understanding adolescent mental health. Adolescents in the low depression–moderate well-being profile (Class 1) reported low levels of depressive symptoms but only moderate well-being, suggesting that the absence of marked distress does not necessarily indicate optimal positive functioning. This group might therefore be overlooked if the assessment focuses only on psychopathological symptoms. Conversely, adolescents in the moderate depression–moderate well-being profile (Class 3) reported moderate depressive symptoms while still maintaining moderate well-being, indicating that psychological distress and positive functioning can coexist. These findings are consistent with the profile patterns identified in the LPA and support the value of the dual-factor model for capturing heterogeneity in adolescent mental health.

PSU differed significantly across the four mental health profiles, supporting H2. Specifically, PSU was lowest in the very low depression–high well-being profile and highest in the high depression–low well-being profile, suggesting a graded association between PSU and poorer mental health. This pattern is consistent with previous evidence showing that PSU is associated with poorer psychological adjustment, including more severe depressive symptoms and lower well-being (Coyne et al., 2019; Mahapatra, 2019; Wang et al., 2025). One possible interpretation is that PSU might be associated with experiences less supportive of adolescents’ needs for autonomy, competence and relatedness. For example, compulsive and dysregulated smartphone use might be less consistent with autonomy because such use is not fully self-endorsed. It might also be associated with reduced competence by disrupting attention, academic engagement and task performance (Paterna et al., 2024). Previous evidence that greater daily smartphone use is associated with poorer academic ranking is consistent with this interpretation (Felisoni and Godoi, 2018). In addition, PSU might be associated with fewer opportunities for high-quality face-to-face interaction, and therefore with weaker relatedness (Coyne et al., 2019). Greater exposure to idealised online content might also coincide with upward social comparison, which might be associated with lower subjective well-being.

EGM statistically mediated the associations of PSU with well-being and depressive symptoms, supporting H3. Given the cross-sectional design, this mediating pattern should be interpreted as an indirect statistical association rather than evidence of a confirmed causal mechanism. One possible explanation is that PSU might be linked to patterns of cognitive and emotional engagement that are less supportive of adaptive emotion regulation. Frequent and dysregulated smartphone use might be associated with greater attentional demands and greater difficulty engaging in reflective emotion regulation (Sweller, 2010). Under such conditions, adolescents might rely more often on less adaptive strategies, such as suppression or avoidance (Elhai et al., 2018). Repeated difficulties in managing negative emotions might, in turn, be associated with weaker beliefs that emotions are controllable and changeable, and with a lower EGM (Schroder et al., 2015). Because a weaker EGM has been associated with greater helplessness, more depressive symptoms and lower well-being (Kneeland and Dovidio, 2020), it could be a meaningful variable for future longitudinal and intervention research. However, the indirect effects observed in this study were modest, suggesting that an EGM is likely to represent only one of several factors involved in the PSU–adolescent mental health association.

The moderation analyses further showed that the negative PSU–EGM association was stronger among students with LBE, supporting H4. Although the interaction effect was modest in magnitude, the simple slope analyses indicated that the negative association was slightly stronger among students with LBE than among those without such experience. The conditional indirect effects with EGM as a statistical mediator were also somewhat larger in the left-behind group, suggesting that family context might be relevant to adolescents’ susceptibility to the psychological correlates of PSU. One possible explanation is that adolescents with LBE might have fewer consistent opportunities for everyday emotional guidance from parents or primary caregivers, a view that is consistent with evidence linking limited parental involvement and family support with depression and anxiety among rural children and adolescents (Fellmeth et al., 2018; Jiang et al., 2024). Under such conditions, PSU might be more strongly associated with difficulties in maintaining the belief that emotions can be changed and regulated through effort. It should also be noted that LBE in this study was assessed as a broad family-context indicator rather than as a uniform developmental condition. The experiences captured by this variable might vary substantially in terms of timing, duration, caregiving arrangements and emotional support, which might partly explain why the observed moderating effect was statistically reliable but modest in size. Accordingly, the LBE should be considered a relevant contextual factor in future research on adolescent PSU and mental health. However, given the cross-sectional design and the modest size of the interaction, these findings should be interpreted cautiously and examined further in longitudinal and intervention studies.

Several limitations of this study should be noted. First, although the cross-sectional design enabled this study to examine associations among PSU, EGM, LBE and mental health in a large sample, it did not allow conclusions about temporal ordering. Future longitudinal research could clarify how these variables are related to one another over time. Second, all main variables were assessed using self-report questionnaires at the same time point. Although Harman’s single-factor test did not indicate substantial common method bias, it cannot be ruled out. Future studies could incorporate objective indicators, such as screen-time records, and reports from multiple informants. Third, participants were recruited from several schools in a single city in China, and students were nested within classes and schools. Future research could use multilevel modelling or cluster-robust standard errors and replicate the findings in more diverse contexts. Fourth, the LBE was assessed using a single retrospective item, which provided a practical indicator of early family context but did not capture detailed information about the timing, duration or caregiving arrangements associated with parental absence. Finally, although several covariates were considered, residual confounding cannot be ruled out. Unmeasured factors such as family functioning, parenting quality, academic stress or pre-existing psychological vulnerability might also have contributed to the observed associations.

Overall, this study extends the application of the dual-factor model of mental health to Chinese senior high school students and highlights EGM and LBE as relevant factors in the PSU–adolescent mental health association. The findings suggest that adolescent mental health in the digital era should be understood not only in terms of psychopathological symptoms, but also in relation to positive functioning, emotional beliefs and family context. At the same time, given the cross-sectional design, the practical implications of these findings should be regarded as preliminary and examined further in longitudinal and intervention studies.

## Conclusion

This study applied a dual-factor model of mental health to a large school-based sample of Chinese senior high school students and identified meaningful heterogeneity in the coexistence of depressive symptoms and well-being. PSU varied systematically across the identified mental health profiles, with higher levels observed in profiles characterised by greater depressive symptoms and lower well-being. EGM was statistically consistent with an indirect association between PSU and well-being and depressive symptoms, whereas LBE shaped the strength of the PSU–EGM association.

More broadly, these findings highlight the value of understanding adolescent mental health in the digital era from a multidimensional and context-sensitive perspective. Assessment and future prevention-oriented research should consider not only psychopathological symptoms, but also positive functioning, emotional beliefs, and family context. Given the cross-sectional and self-report nature of this study, the practical implications should be interpreted cautiously and further examined through longitudinal and intervention-oriented research.

## Supporting information

10.1017/gmh.2026.10247.sm001 Mao et al. supplementary material Mao et al. supplementary material

## Data Availability

The data that support the findings of this study are available from the corresponding author (LW) upon reasonable request.

## Supplementary material

The supplementary material for this article can be found at http://doi.org/10.1017/gmh.2026.10247.

## Inclusivity statement

The study sample included a large proportion of left-behind adolescents from rural China, a historically underrepresented group in mental health research. The research team consists of both senior and early-career researchers: one master’s supervisor and three master’s students in psychology, all based in China, with direct knowledge of the local educational and family contexts. The authors have cited relevant literature from low- and middle-income countries alongside global studies. The survey instruments were validated in Chinese adolescent populations and administered in the participants’ first language. No exclusion was applied based on gender, school type or family structure, and participation was voluntary for all students.

## Long descriptions

### Table 1. Long description

Table 1 compares fit indices across 1 to 4 latent classes. Columns: No. of Classes, AIC, BIC, aBIC, LMRT p-value, BLRT p-value, Entropy, Class Probabilities (%). For 4 classes (bold): AIC = 1,172,222.08, BIC = 1,172,924.12, aBIC = 1,172,660.34, LMRT p < 0.001, BLRT p < 0.001, Entropy = 0.955, class probabilities = 34.44%/52.59%/9.37%/3.60%. Despite the smallest class being 3.60%, it was retained for distinct psychological profile and model stability. For 2 classes: entropy = 0.927; 3 classes: entropy = 0.912. AIC/BIC/aBIC decrease progressively from 1 to 4 classes.

Navigate back to Table 1..

### Figure 1. Long description

A line graph and data table titled Latent Profiles of Depression and Well-being (n = 34,831). The y-axis represents the Mean score from 0.0 to 4.0. The x-axis lists 15 indicators: happy, relax, vitality, adequate sleep, interesting, low spirits, impatient, poor sleep, waning interest, fatigue, inattentive, worthlessness, dull, anxious, feeling unwell, and self-harm.

Four classes are plotted:

- Class 1 (low depression-moderate well-being, 34.44%): Starts with moderate scores (2.769 for happy) and drops sharply after ‘interesting’ to low scores (0.048 for self-harm).

- Class 2 (very low depression-high well-being, 52.59%): Starts with the highest scores (4.012 for happy) and drops to the lowest scores among all groups for negative indicators (0.028 for self-harm).

- Class 3 (moderate depression-moderate well-being, 9.37%): Maintains mid-range scores across all indicators, ending at 0.297 for self-harm.

- Class 4 (high depression-low well-being, 3.60%): Starts with the lowest scores for positive indicators (1.592 for happy) but rises to the highest scores for negative indicators, peaking at 2.074 for fatigue and ending at 1.554 for self-harm.

A crossover occurs between the ‘interesting’ and ‘low spirits’ indicators, where the high well-being groups (Classes 1 and 2) drop below the high depression group (Class 4).

Navigate back to Figure 1..

### Table 2. Long description

Table 2 presents means and standard errors of Problematic Smartphone Use (PSU) and Emotional Growth Mindset (EGM) across four latent classes. For PSU: Class 2 (very low depression–high well-being) = 1.82 ± 0.007; Class 1 (low depression–moderate well-being) = 2.36 ± 0.010; Class 3 (moderate depression–moderate well-being) = 3.02 ± 0.012; Class 4 (high depression–low well-being) = 3.57 ± 0.025. Overall χ2(3) = 10,729.47, p < 0.001. Post-hoc: C2 < C1 < C3 < C4. For EGM: Class 2 = 4.10 ± 0.008; Class 1 = 3.83 ± 0.009; Class 3 = 3.41 ± 0.010; Class 4 = 2.95 ± 0.023. Overall χ2(3) = 4,326.45, p < 0.001. Post-hoc: C4 < C3 < C1 < C2. All pairwise comparisons p < 0.001.

Navigate back to Table 2..

### Figure 2 Long description

Figure 2 illustrates the mediation model. Variables: Problematic Smartphone Use (PSU), Emotional Growth Mindset (EGM), Well-being, Depression. Standardised coefficients: PSU → EGM = −0.12; EGM → Well-being = 0.26; EGM → Depression = −0.34. Direct effects: PSU → Well-being = −0.26; PSU → Depression = 0.31. Indirect effects (via EGM): PSU → Well-being = −0.032 (10.85% of total effect −0.37); PSU → Depression = 0.041 (11.68% of total effect 0.45). All coefficients p < 0.001. Covariates (age, gender, grade, school type, family structure, left-behind experience) are controlled but not shown.

Navigate back to Figure 2.

### Table 3. Long description

The table is organized into three main outcome columns: Emotional growth mindset, Well-being, and Depression. Each outcome is evaluated by beta, S E, and t values.

* Problematic smartphone use: For Emotional growth mindset, beta is minus 0.12, S E is 0.002, t is minus 51.33. For Well-being, beta is minus 0.26, S E is 0.003, t is minus 74.72. For Depression, beta is 0.31, S E is 0.003, t is 98.60.

* Emotional growth mindset: For Well-being, beta is 0.26, S E is 0.008, t is 33.61. For Depression, beta is minus 0.34, S E is 0.007, t is minus 48.72.

* Left-behind experience: For Emotional growth mindset, beta is 0.35, S E is 0.04, t is 8.67.

* P S U times Left-behind experience: For Emotional growth mindset, beta is minus 0.02, S E is 0.005, t is minus 3.41.

* Model Statistics: F values are 461.97 for Emotional growth mindset, 1,652.26 for Well-being, and 2,826.54 for Depression. R-squared values are 0.09, 0.22, and 0.33 respectively.

All t values are significant at p < 0.001.

Navigate back to Table 3..

### Figure 3 Long description

Figure 3 displays the moderating effect of left-behind experience (LBE) on the PSU–EGM relationship. X-axis: Problematic Smartphone Use (low to high). Y-axis: Emotional Growth Mindset. Two lines: without LBE (LBE = 0) slope β_simple = −0.11 (p < 0.001); with LBE (LBE = 1) slope β_simple = −0.13 (p < 0.001). The negative association is stronger among students with LBE. Interaction term PSU × LBE: β = −0.02 (p < 0.001).

Navigate back to Figure 3.
