# Supplementary material for: Problematic smartphone use and adolescent mental health: The mediating role of emotional growth mindset and the moderating role of left-behind experience
Source: Glob Ment Health (Camb). 2026 Jun 15;13:e127. doi: 10.1017/gmh.2026.10247 (PMC13312359; doi:10.1017/gmh.2026.10247)
Supplement: Mao et al. supplementary material [file S2054425126102477sup001.docx]

**Scale 1. World Health Organization Well-Being Index (WHO-5)**

Please carefully reflect on the past two weeks and select the statement below that most closely describes your state of mind.

| WHO-5 | All times | Most of the time | more than half the time | Less than half the time | Sometimes | never |
| --- | --- | --- | --- | --- | --- | --- |
| I feel happy and content. | 0 | 1 | 2 | 3 | 4 | 5 |
| I feel calm and relaxed. | 0 | 1 | 2 | 3 | 4 | 5 |
| I feel full of energy and vitality. | 0 | 1 | 2 | 3 | 4 | 5 |
| Upon waking, I felt refreshed and had adequate rest. | 0 | 1 | 2 | 3 | 4 | 5 |
| My daily life is filled with interesting things. | 0 | 1 | 2 | 3 | 4 | 5 |

**Scale 2. Kutcher Adolescent Depression Scale (KADS-11)**

Please carefully reflect on the past month and select the answer that best applies to you, marking an “√” next to the corresponding number. Only one answer may be chosen per question.

| KADS-11 | never | On many occasions | Most of the time | at all times |
| --- | --- | --- | --- | --- |
| Feel low spirits | 0 | 1 | 2 | 3 |
| Feel impatient | 0 | 1 | 2 | 3 |
| Experiencing sleep difficulties | 0 | 1 | 2 | 3 |
| Waning interest | 0 | 1 | 2 | 3 |
| Feel fatigue | 0 | 1 | 2 | 3 |
| Feel inattentive | 0 | 1 | 2 | 3 |
| Feel of worthlessness | 0 | 1 | 2 | 3 |
| Finding life dull | 0 | 1 | 2 | 3 |
| Prone to worry and anxiety | 0 | 1 | 2 | 3 |
| Feeling unwell | 0 | 1 | 2 | 3 |
| Thoughts and actions that harm oneself | 0 | 1 | 2 | 3 |

**Scale 3. Problematic Smartphone Use Scale Short (PMPUS-S)**

Select the most appropriate response from the following options and mark the corresponding box with a tick “√”. Choose only one option per question.

| PMPUS-S | highly inconsistent | Not quite fitting | Uncertain | more in line with | highly suitable |
| --- | --- | --- | --- | --- | --- |
| When my mobile phone is not to hand and I cannot tell whether someone might be trying to contact me, I feel quite anxious. | 1 | 2 | 3 | 4 | 5 |
| When my mobile phone is not to hand and I cannot receive text messages or calls, I feel anxious. | 1 | 2 | 3 | 4 | 5 |
| When my mobile phone is not to hand, preventing me from checking my QQ, WeChat, emails and other messages, I feel anxious. | 1 | 2 | 3 | 4 | 5 |
| When my mobile phone is not to hand and I find myself disconnected from the internet, I become quite anxious. | 1 | 2 | 3 | 4 | 5 |
| If I go without my mobile phone for a while, I feel uneasy. | 1 | 2 | 3 | 4 | 5 |
| My mobile phone is an integral part of my life; if it's not within reach, I feel restless and uneasy. | 1 | 2 | 3 | 4 | 5 |
| If I cannot use my mobile phone for a period of time, I feel quite anxious. | 1 | 2 | 3 | 4 | 5 |
| When my mobile phone has no signal or cannot access the internet, I feel uneasy and irritable. | 1 | 2 | 3 | 4 | 5 |

**Scale 4. Theories of Emotion Scale (TOE).**

Please answer according to your own views and tick the corresponding number. Select one answer per question.

| TOE | Completely inconsistent | Not quite fitting | A bit out of place | Somewhat fits the bill | more in line with | highly suitable |
| --- | --- | --- | --- | --- | --- | --- |
| Everyone can learn to control their emotions. | 1 | 2 | 3 | 4 | 5 | 6 |
| If they want to, people can change the emotions that they have. | 1 | 2 | 3 | 4 | 5 | 6 |
| No matter how hard they try, people can’t really change the emotions that they have. | 1 | 2 | 3 | 4 | 5 | 6 |
| The truth is, people have very little control over their emotions. | 1 | 2 | 3 | 4 | 5 | 6 |

**Scale 5. Left-Behind Experience (LBE)**

| Experience of Left-Behind | No | Yes |
| --- | --- | --- |
| Were important family members (father or mother) frequently absent from your life for more than six months before the age of six? | 0 | 1 |

**Table S1**

Sociodemographic Characteristics of the Participants (n = 34,831)

| **Variable** | ***n*** | **%** | **Variable** | ***n*** | **%** |
| --- | --- | --- | --- | --- | --- |
| **Left-behind Experience** |  |  | **Grade** |  |  |
| Non-left-behind | 12768 | 36.7 | Grade 10 | 13870 | 39.8 |
| Left-behind | 22063 | 63.3 | Grade 11 | 11773 | 33.8 |
| **Gender** |  |  | Grade 12 | 9188 | 26.4 |
| Male | 20095 | 57.7 | **Family Structure** |  |  |
| Female | 14736 | 42.3 | Intact Family | 30403 | 87.3 |
| **School Type** |  |  | Single-parent | 1943 | 5.6 |
| Public | 22993 | 66 | Blended Family | 2324 | 6.7 |
| Private | 11838 | 34 | Orphan | 161 | 0.5 |

**Table S2.** Correlation Matrix of Key Variables.**p* < 0.05, ***p* < 0.01, ***p* < 0.001. The same applies below

| **Variable** | ***M*** | ***SD*** | **1** | **2** | **3** | **4** |
| --- | --- | --- | --- | --- | --- | --- |
| 1.Problematic Smartphone Use | 2.338 | 1.045 | 1 |  |  |  |
| 2. Emotional Growth Mindset | 3.807 | 0.937 | -0.275^**^ | 1 |  |  |
| 3. Well-being | 2.775 | 1.186 | -0.430^**^ | 0.274^**^ | 1 |  |
| 4. Depression | 0.541 | 0.522 | 0.526^**^ | -0.355^**^ | -0.602^**^ | 1 |
