# Supplementary material for: Problematic smartphone use and adolescent mental health: The mediating role of emotional growth mindset and the moderating role of left-behind experience
Source: Glob Ment Health (Camb). 2026 Jun 15;13:e127. doi: 10.1017/gmh.2026.10247 (PMC13312359; doi:10.1017/gmh.2026.10247)
Supplement: Peer review history

# Reviewer report: Review: Problematic smartphone use and adolescent mental health: The mediating role of emotional growth mindset and the moderating role of left-behind experience — R0/PR2

**DOI:** 10.1017/gmh.2026.10247.pr2

[1] Although the study is cross-sectional, the manuscript repeatedly uses causal or mechanistic language, for example stating that PSU “affects” mental health, that harm “operates through” EGM, and that schools can intervene based on these findings because emotion mindset is “teachable.” Similar causal framing also appears in the abstract, conclusion, and impact statement. These claims go beyond what can be supported by a cross-sectional design and mediation analysis. The manuscript should consistently use associational language, such as “is associated with,” “is linked to,” or “is statistically consistent with.” The impact statement in particular is currently too strong for an observational study and should be rewritten more cautiously.

[2] The mediation and moderated mediation analyses are reported as controlling only for age and gender. However, the dataset also includes other potentially relevant variables, including grade, school type, family structure, and left-behind experience itself, and the sample description shows substantial heterogeneity across these characteristics. It is not clear why only age and gender were retained in the main PROCESS models. This raises concern about omitted-variable bias and residual confounding. The authors should provide a theory-driven justification for covariate selection and, ideally, conduct sensitivity analyses with a broader set of covariates. In addition, because this is a school-based survey from a single city, the authors should clarify whether clustering at the school or classroom level was accounted for. If not, this limitation should be acknowledged explicitly.

[3] Table 1 reports model fit indices for one- through four-class solutions, with the four-class model selected as optimal. However, the rationale for stopping at four classes is not sufficiently explained, and the manuscript would benefit from fuller reporting of class selection criteria, theoretical interpretability, class separation, and class assignment quality. I also recommend reporting class-specific means or standardized scores for the LPA indicators in a table, not only in the figure, to aid interpretation. Most importantly, the manuscript currently states that MANOVA on well-being and depression across the latent classes demonstrates “robust discriminant validity.” Because the classes were defined using well-being and depression, testing differences on those same indicators is tautological and should not be interpreted as evidence of discriminant validity. That section should be revised accordingly.

[4] According to the results, PSU scores ranked from highest to lowest as follows: fully pathological, fully healthy, partially healthy, and partially pathological. Likewise, EGM was reportedly highest in the partially mentally healthy group rather than in the fully mentally healthy group. These patterns are not intuitive and may reflect a scoring issue, a labeling problem, or a more complex substantive pattern that requires careful explanation. At minimum, the authors should verify the scoring and class labels, provide descriptive statistics for PSU and EGM by class, and offer a clearer interpretation of why the “fully healthy” group would have higher PSU than two intermediate groups. Without this clarification, readers may question the internal coherence of the class solution.

[5] LBE is measured using a single retrospective yes/no item asking whether a parent was frequently absent for more than six months before age six. This is a very limited operationalization of a complex developmental exposure. It does not capture timing beyond age six, duration, number of absent caregivers, current status, caregiving arrangement, quality of contact, or chronicity of separation. Given that LBE is central to the moderated mediation model, the authors should justify this measure more carefully and expand the limitations section to acknowledge that this single-item measure may not adequately represent the heterogeneity of left-behind experience.

[6] The manuscript relies on Harman’s single-factor test and concludes that there is no severe common method bias because the first factor explains 31% of the variance. This test is widely considered insufficient on its own. The authors should avoid making a strong claim that common method bias is not a concern. Instead, they should state more cautiously that Harman’s test did not indicate severe bias, while acknowledging that self-report, same-source, same-time measurement remains an important limitation. This is especially relevant because all core constructs were measured by self-report in the same survey.

[7] The discussion does a reasonable job summarizing the findings, but parts of the interpretation still extend beyond the analytic design. For example, the manuscript presents EGM as an “important intervention target” and suggests that school-based emotion mindset training could compensate for parental absence. Such statements are interesting hypotheses, but they should be framed explicitly as implications for future intervention research rather than as practice recommendations already supported by the present study. The same caution applies to the concluding section.

# Reviewer report: Review: Problematic smartphone use and adolescent mental health: The mediating role of emotional growth mindset and the moderating role of left-behind experience — R0/PR3

**DOI:** 10.1017/gmh.2026.10247.pr3

General comments:

The aim of this manuscript was to identify latent profiles of mental health among Chinese senior high school students. Although this article addresses an interesting topic, there are some issues should be addressed before publication. Firstly, I suggest to send this manuscript for English language proofreading. Second, there is some inconsistency in spacing before in-text citations (e.g., some references are preceded by a space, while others are not). Please ensure consistent formatting throughout the manuscript.

Specific comments

Introduction

- Page 11, line 36, please indicate the corresponding reference for the statistics.

- Page 11, line 49, Please elaborate more specifically on how problematic mobile phone use influences each of the three basic psychological needs (autonomy, competence, and relatedness)

- Page 11, line 61-64, This sentence is quite long and complex. Consider breaking it into shorter sentences to improve clarity and readability.

- Page 12, line 86, There is some inconsistency in punctuation (e.g., use of non-English punctuation in some instances). Please ensure that all punctuation follows standard English conventions throughout the manuscript.

- Page 12, line 89-101, Please also provide a clearer rationale for using latent profile analysis in the introduction, including why this approach is appropriate for the research questions.

Method

- Page 12, line 89-110, Please clarify the participant recruitment procedures and report the valid response rate, including how the final analytic sample was derived.

- In the Measures section, please briefly clarify the purpose of each scale (i.e., what construct each instrument is intended to assess)

- Please provide more detail in the Data Analysis section. For example, clarify which model fit indices were used to evaluate the LPA models (e.g., AIC, BIC, aBIC, or entropy) and the criteria for selecting the final model.

Results

- Some methodological details related to the statistical analysis (e.g., criteria for selecting the optimal number of latent classes) are currently presented in the Results section. These should be moved to the Data Analysis (Methods) section to improve clarity and adhere to standard reporting structure.

- Page 15, line 192-196, Please provide clearer definitions of the latent profile labels. Specifically, indicate what each group represents in terms of the combination and range of scores on scales, so that readers can better interpret the profiles. In addition, the current labels (e.g., ‘partially healthy,’ ‘fully pathological’) may be misleading. According to contemporary perspectives (Galderisi et al., 2015), mental health is not simply the absence or presence of symptoms, but a dynamic state of balance. Therefore, I recommend refining the group labels to more accurately reflect the underlying score patterns and to avoid potentially stigmatizing or oversimplified terminology.

Galderisi S, Heinz A, Kastrup M, Beezhold J, Sartorius N. Toward a new definition of mental health. World Psychiatry. 2015 Jun;14(2):231-3. doi: 10.1002/wps.20231. PMID: 26043341; PMCID: PMC4471980.

Conclusion

- The conclusion currently repeats the results; please revise it to better synthesize the findings and highlight their broader implications and significance.

# Reviewer report: Review: Problematic smartphone use and adolescent mental health: The mediating role of emotional growth mindset and the moderating role of left-behind experience — R0/PR4

**DOI:** 10.1017/gmh.2026.10247.pr4

1. Introduction

• The manuscript alternates between: digital technology use, mobile phone use, problematic mobile phone use, and smartphone use. This creates conceptual ambiguity. Please clarify whether these terms are interchangeable or distinct constructs as well as clearly define and consistently use one core term (preferably aligned with the title, e.g., problematic smartphone use)

• Multiple theories are introduced, but their integration is somewhat fragmented rather than synthesized. Please explicitly explain how these theories jointly support the model. For example (lines 49-58), the authors stated that problematic mobile phone use can be part of Self-Determination Theory. However, this is not the case as the Self-Determination Theory does not mention anything on problematic use.

• Line 82: Please define “left-behind adolescents” explicitly. The concept is important but context-specific (China), please emphasize its theoretical relevance beyond China (e.g., parental absence, migration contexts)

• Careful proofreading is needed. There are multiple instances of missing spaces before in-text citations and minor punctuation inconsistencies throughout the manuscript. These issues affect readability and should be carefully corrected.

• Line 43: outcomes(Mansfield et al. 2025).

• Line 48: outcomes(NASEM,2024), (NASEM,2024) missing space, lack of period in the end of paragraph

• Line 54: nature(Coyne et al. 2019; Paterna et al. 2024)

• Line 56: well-being(Coyne et al. 2019; Mahapatra 2019; Wang et al. 2025)

• Line 67: regulation(Sweller 2010)

• Line 72: multitasking(Wickord and Quaiser-Pohl, 2025)

• Line 74: reappraisal(J. D. Elhai et al. 2017)

• Line 75: controllability(Ford and Gross 2018)

• Line 79: capital(Xiong et al. 2020; Zhang et al. 2023)

• Line 81: development(Bronfenbrenner 1979).

• Line 86: “adolescent’capacity” missing “s”

• Line 88: depleted(Zhen et al. 2020)

2. Methods

• This section requires substantial revision for clarity, completeness, and methodological rigor, particularly regarding sampling procedures and measurement validity.

• The manuscript reports a very large sample (n = 34,831) but does not explain sampling method, recruitment procedures, geographic location(s), inclusion/ exclusion criteria, ethical approval, and consent procedures.

• Line 104: The manuscript refers to “valid responses” but does not explain the criteria for validity and data cleaning procedures. Please clarify how invalid responses were identified and removed, and how missing data was handled.

• The manuscript does not clearly specify which scale corresponds to each study variable. Please explicitly indicate which instruments were used to measure each construct.

• Insufficient detail on measurement validity, while Cronbach’s α values are reported, the manuscript lacks evidence of construct validity and whether the Chinese versions are validated. For each scale, please indicate validation in Chinese adolescent populations.

• KADS-11 description lacks the number of items.

• Left-behind experience measurement is overly simplistic. Measured using a single yes/no item, which may oversimplify a complex construct and introduce recall bias (before age six).

• Many formatting and spacing issues

3. Results + Discussion

• It is not clear whether the LPA was conducted using item-level indicators or composite scores of well-being and depression. Please clarify the indicators included in the model.

• The rationale for selecting only well-being and depression variables as indicators for LPA could be further elaborated. Additional explanation would help readers understand how these indicators sufficiently capture the heterogeneity of adolescent mental health.

• Lines 263-264: The manuscript implies causal relationships, for example: “problematic smartphone use may impair mental health”. The study is cross-sectional, so causal interpretations are not warranted. The interpretation of findings should be more cautious. Some statements imply causal relationships (e.g., impair), which would be better reframed as associations.

• The Discussion would benefit from greater consideration of the practical significance of the findings, particularly for the moderation and mediation effects, which appear relatively small in magnitude despite statistical significance.

• The proposed mediating mechanism involving an emotional growth mindset would benefit from clearer and more explicit theoretical integration, particularly in explaining how cognitive processes translate into changes in emotional belief systems.

• The interpretation of left-behind experience would benefit from further clarification and stronger linkage to the reported results, particularly regarding baseline differences in emotional growth mindset.

• Inconsistent use of: “partial mental health” vs “partially mentally healthy”, “complete mental health” vs “fully healthy”

• Limitations section is appropriate but could also mention: Simplified measurement of left-behind experience and potential residual confounding.

# Editor report: Recommendation: Problematic smartphone use and adolescent mental health: The mediating role of emotional growth mindset and the moderating role of left-behind experience — R0/PR5

**DOI:** 10.1017/gmh.2026.10247.pr5

**Affiliations:** Shenzhen University, China

No accompanying comment.

# Editor report: Decision: Problematic smartphone use and adolescent mental health: The mediating role of emotional growth mindset and the moderating role of left-behind experience — R0/PR6

**DOI:** 10.1017/gmh.2026.10247.pr6

**Affiliations:** London School of Hygiene & Tropical Medicine, United Kingdom of Great Britain and Northern Ireland

No accompanying comment.

# Editor report: Recommendation: Problematic smartphone use and adolescent mental health: The mediating role of emotional growth mindset and the moderating role of left-behind experience — R1/PR8

**DOI:** 10.1017/gmh.2026.10247.pr8

**Affiliations:** Shenzhen University, China

Thanks for addressing the questions raised by two reviewers. Now I would like to make an acceptance recommendation. Liye

# Editor report: Decision: Problematic smartphone use and adolescent mental health: The mediating role of emotional growth mindset and the moderating role of left-behind experience — R1/PR9

**DOI:** 10.1017/gmh.2026.10247.pr9

**Affiliations:** London School of Hygiene & Tropical Medicine, United Kingdom of Great Britain and Northern Ireland

No accompanying comment.
